# Glycolytic cancer-associated fibroblasts orchestrate metabolic remodeling in the tumor microenvironment

**DOI:** 10.1186/s13046-026-03756-w

**Published:** 2026-06-11

**Authors:** Xinyu Cui, Akshaya Poonepalle, Chloe Shay, Yong Teng

**Affiliations:** 1 Department of Hematology and Medical Oncology, Winship Cancer Institute, Emory University, Atlanta, GA 30322 USA; 2 Wallace H. Coulter Department of Biomedical Engineering, Georgia Institute of Technology and Emory University, Atlanta, GA 30322 USA

**Keywords:** GlyCAFs, Metabolic heterogeneity, The tumor microenvironment, The glyCAF-tumor axis, Co-dependencies

## Abstract

Cancer-associated fibroblasts (CAFs) are key regulators of the tumor microenvironment and are predominantly classified according to transcriptional identity. However, this framework overlooks an important dimension of CAF biology, metabolic heterogeneity, which remains insufficiently characterized and is not incorporated into current classification systems. In this review, we advance the concept of glycolytic cancer-associated fibroblasts (glyCAFs) as a metabolically defined stromal subset that functions as a central organizer of the tumor ecosystem. Integrating insights from metabolic profiling, transcriptomics, spatial analyses, and emerging therapeutic studies, we outline a model in which glyCAFs coordinate tumor progression through three interconnected mechanisms: metabolic support of oxidative tumor cells via lactate shuttling, suppression of antitumor immunity through extracellular acidification, and spatial organization of the invasive niche through structured stromal barriers. We further discuss evidence from solid tumors supporting the broader relevance of glyCAF-driven metabolic coupling as a conserved feature of tumor biology. Importantly, we highlight targetable vulnerabilities within the glyCAF-tumor axis, including pathways currently under clinical investigation, and consider the potential of glyCAF abundance as a biomarker of therapeutic response, particularly to immune checkpoint blockade. By integrating metabolic state as an orthogonal axis of CAF identity, we suggest a revised framework for tumor stratification and highlight new opportunities to disrupt tumor-stroma co-dependencies through metabolic-immunologic combination therapies.

## Introduction

 Cancer-associated fibroblasts (CAFs) constitute the dominant non-immune stromal population in many solid tumors, comprising up to 80% of the tumor mass in advanced disease [1]. Far from being passive structural elements, CAFs are dynamic and heterogeneous regulators of tumor progression. They actively shape tumor behavior by secreting growth factors, cytokines, and chemokines that promote cancer cell proliferation and survival, while also remodeling the extracellular matrix to facilitate invasion and metastatic dissemination. In parallel, CAFs contribute to neovascularization by supporting angiogenic programs and modulate therapeutic response by fostering drug resistance through both biochemical and physical mechanisms. Importantly, CAFs exert profound effects on the immune microenvironment, orchestrating immunosuppressive networks that limit effective antitumor immunity [2, 3]. These multifaceted functions position CAFs as central organizers of the tumor ecosystem rather than mere bystanders within it.

Single-cell transcriptomic analyses have uncovered extensive heterogeneity within the CAF compartment of solid tumors, including head and neck squamous cell carcinomas (HNSCC) [4, 5]. These studies have identified multiple CAF subtypes, such as myofibroblastic, inflammatory, antigen-presenting, and extracellular matrix (ECM)-associated populations, each defined by distinct transcriptional programs. Importantly, these subtypes exhibit differential roles in tumor progression, modulation of the immune microenvironment, and responsiveness to immunotherapy, highlighting the functional diversity of CAFs within the tumor ecosystem [6–9]. However, current classification frameworks are defined largely by transcriptional identity, leaving an important dimension underexplored: metabolic state. Glycolytic activity, reflected by metabolic enzyme expression, lactate production, mitochondrial content, and metabolic flux, is not merely a downstream consequence of transcriptional programs but a functional property with direct effects on metabolite availability, extracellular pH, immune cell function, and drug sensitivity. A given CAF subtype may or may not exhibit a glycolytic phenotype, and this metabolic state may be as biologically consequential as its transcriptional classification.

The concept of metabolically reprogrammed stroma is supported by established models of tumor metabolism. While the classical Warburg effect describes aerobic glycolysis in tumor cells [10], the reverse Warburg effect extends this paradigm to the surrounding stroma: tumor cells induce aerobic glycolysis in adjacent fibroblasts, which export lactate through monocarboxylate transporter 4 (MCT4) for subsequent import by oxidative tumor cells through MCT1, creating a metabolically coupled two-compartment system [11, 12]. In HNSCC, immunohistochemical analyses have demonstrated this compartmentalized architecture, with proliferative, mitochondria-rich carcinoma cells located adjacent to glycolytic stromal fibroblasts [13]. Subsequent work has identified tumor-derived signals that induce and maintain this glycolytic phenotype [14, 15], along with environmental factors such as tobacco exposure that further reinforce stromal metabolic reprogramming [16].

These glycolytic fibroblasts, which we term glycolytic cancer-associated fibroblasts (glyCAFs), represent a metabolically defined stromal population that links tumor metabolism, immune regulation, and spatial organization within the tumor microenvironment (TME). We propose glyCAFs as a unifying framework for understanding how stromal metabolism shapes tumor progression, immune evasion, and therapeutic resistance in HNSCC. This review synthesizes metabolic, transcriptomic, spatial, and therapeutic evidence to define glyCAFs at the molecular and functional levels, examine the mechanisms governing their induction and stabilization, delineate their effects on tumor behavior and immune function, and evaluate therapeutic strategies aimed at disrupting glyCAF-tumor metabolic coupling. Converging evidence across multiple solid tumor types further supports the broader relevance of this framework beyond HNSCC.

## GlyCAFs

### Molecular and functional features of glyCAFs

**Fig. 1:**
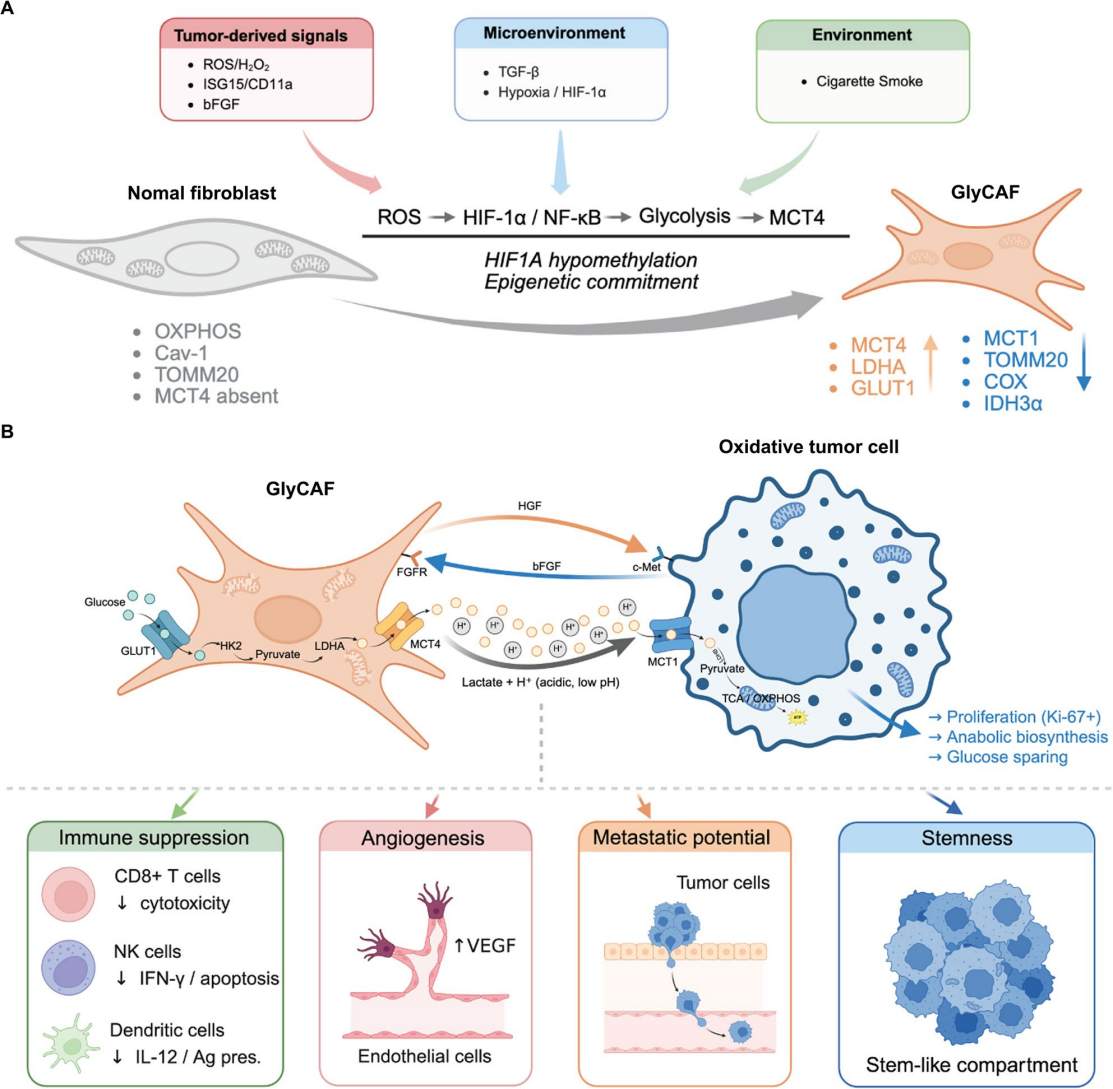
Induction of glyCAFs and their metabolic coupling with tumor cells. **A** Tumor-derived signals, microenvironmental cues, and environmental factors promote the transition of normal fibroblasts into glyCAFs, with increased glycolytic features and MCT4 expression and reduced expression of mitochondrial and oxidative markers. **B** GlyCAFs release lactate into the TME, which is subsequently taken up by oxidative tumor cells to fuel mitochondrial metabolism. This metabolic coupling is accompanied by reciprocal growth factor signaling between glyCAFs and tumor cells, driving downstream processes such as immune suppression, angiogenesis, enhanced metastatic potential, and the maintenance of stem-like tumor cell states

GlyCAFs represent a metabolically specialized subset of CAFs characterized by elevated glycolytic activity and lactate production. This identity is supported by a combination of molecular markers and functional metabolic features. Positive markers include MCT4 (SLC16A3) [17], the principal lactate exporter and a hypoxia-inducible factor 1-alpha (HIF-1α) target [18]; lactate dehydrogenase A (LDHA), which converts pyruvate to lactate; and glucose transporter 1 (GLUT1; SLC2A1), indicating increased glucose uptake [19]. The negative profile further refines this identity: glyCAFs lack MCT1 (SLC16A1), the lactate importer characteristic of oxidative tumor cells; show low expression of mitochondrial markers TOMM20 and cytochrome c oxidase (COX), consistent with reduced mitochondrial content; and exhibit decreased expression of the tricarboxylic acid (TCA) cycle enzyme IDH3α [13, 20]. Functionally, glyCAFs demonstrate high extracellular acidification rate (ECAR) and low oxygen consumption rate (OCR) in Seahorse assays and produce elevated lactate levels, consistent with a glycolytic phenotype [14]. The composite signature MCT4^+^/MCT1^-^/TOMM20-low/COX-low/IDH3α-low can be assessed by immunohistochemistry, flow cytometry, or single-cell transcriptomics to distinguish glyCAFs from quiescent fibroblasts, which are MCT4-negative with intact mitochondrial function, and from oxidative tumor cells, which express MCT1 alongside high mitochondrial activity (Table 1; Fig. 1A). Whether this metabolic identity represents a distinct transcriptional cluster or a functional state that can be adopted across multiple CAF subtypes remains unresolved and will require integrated transcriptomic and metabolic analyses within the same specimens.

**Table 1:** Molecular and functional features of glyCAFs

Category	Marker / Assay	Description
Positive markers	MCT4 (SLC16A3)	Principal lactate exporter; HIF-1α transcriptional target; stromal expression specific to cancer tissue
	LDHA	Converts pyruvate to lactate; terminal glycolytic enzyme
	GLUT1 (SLC2A1)	Glucose transporter; upregulated to increase glucose uptake
Negative markers	MCT1 (SLC16A1)	Absent; high-affinity lactate importer expressed on oxidative tumor cells
	TOMM20	Low expression; reflects mitochondrial depletion
	COX	Low activity: cytochrome c oxidase, indicates suppressed OXPHOS
	IDH3α	Reduced expression; TCA cycle enzyme marking glycolytic shift
Functional readouts	ECAR	High ECAR on Seahorse analysis
	OCR	Low OCR on Seahorse analysis ; reduced mitochondrial respiration
	Extracellular lactate	Elevated lactate in conditioned media
Composite signature	MCT4^+^ / MCT1^−^ / TOMM20-low / COX-low / IDH3α-low	Proposed immunohistochemical panel for glyCAF identification in clinical specimens

### In-patient validation

The molecular definition of glyCAFs is supported by direct evidence from HNSCC patient tissues. Curry et al. performed a systematic immunohistochemical analysis of metabolic compartments, identifying proliferative, mitochondria-rich carcinoma cells (Ki-67^+^, TOMM20^+^, COX^+^, MCT1^+^), non-proliferative carcinoma cells, and non-proliferative stromal fibroblasts [13]. MCT4 was highly specific to tumor tissues and absent from adjacent normal mucosa, and within tumors, stromal MCT4 marked cancer-associated fibroblasts (*p* < 0.001). These MCT4-positive CAFs were consistently MCT1-negative, indicating lactate export rather than import. Stromal MCT4 expression correlated with higher tumor stage (*p* < 0.03), while epithelial MCT4 in non-proliferative carcinoma cells predicted recurrence (*p* < 0.0001) and associated with fluorodeoxyglucose positron emission tomography (FDG-PET) avidity (*p* < 0.04). Domingo-Vidal et al. further demonstrated that MCT4 upregulation with concurrent IDH3α downregulation defines glycolytic CAFs, whereas MCT1 and TOMM20 characterize oxidative tumor cells [20]. Genetic evidence was provided by Bisetto et al., showing that MCT4-knockout mice exposed to 4-nitroquinoline 1-oxide developed significantly reduced oral squamous cell carcinoma compared to wild-type controls, supporting a functional role for stromal glycolysis in tumor development [21]. Together, these findings establish glyCAFs as a definable stromal population in HNSCC, with molecular, clinical, and genetic evidence supporting their contribution to disease progression.

## Induction and stabilization of the GlyCAF phenotype

### Mechanisms of glycolytic induction

The conversion of quiescent fibroblasts into glyCAFs is driven by converging inputs from tumor-derived factors, microenvironmental stress, and environmental exposures (Fig. 1A) [16, 22, 23]. Although first described in breast cancer, the reverse Warburg effect is also relevant in HNSCC, where glyCAF induction is linked to a reactive oxygen species (ROS)- HIF-1α / NF-κB-glycolysis-MCT4 cascade (Fig. 1A) [11, 18, 24].

Among tumor-derived signals, Wang et al. identified interferon-stimulated gene 15 (ISG15) as a secreted factor that recruits fibroblasts into the TME and promotes glycolytic reprogramming in oral squamous cell carcinoma [15]. Tumor-derived ISG15 binds the lymphocyte function-associated antigen 1 (LFA-1, also known as CD11a) receptor on fibroblasts, driving both glycolytic conversion and fibroblast migration toward the tumor. Tumors with high ISG15 expression showed increased collagen deposition, alpha-smooth muscle actin (α-SMA) expression, and fibroblast activation protein, consistent with enhanced CAF recruitment and activation. Reactive oxygen species represent another major class of tumor-derived signals. In the original reverse Warburg model, tumor cell-derived hydrogen peroxide induces oxidative stress in fibroblasts, stabilizing HIF-1α under normoxic conditions and activating NF-κB to drive MCT4 expression. Loss of Caveolin-1 in fibroblasts is a critical event in this process, promoting mitochondrial dysfunction and glycolytic conversion.

Environmental exposure provides a third induction pathway that is particularly relevant in HNSCC. Domingo-Vidal et al. showed that cigarette smoke extract induces oxidative stress, glycolytic flux, MCT4 expression, and senescence-associated features in fibroblasts [16]. Smoke-exposed fibroblasts displayed increased lactate secretion, and co-culture and co-injection experiments confirmed that they promoted more aggressive tumor behavior than unexposed controls. Notably, MCT4 knockout fibroblasts were dramatically more sensitive to cigarette smoke-induced apoptosis, with approximately 90% higher death rates than wild-type fibroblasts [16], suggesting that MCT4 is not only a marker of glycolytic reprogramming but also a survival mechanism that protects glyCAFs from oxidative injury. These findings directly link the dominant environmental risk factor for HNSCC to induction of the glyCAF phenotype. Although the evidence above pertains primarily to HNSCC, glycolytic induction may also become epigenetically durable. In breast cancer models, Becker et al. showed that chronic hypoxia induces HIF1A promoter hypomethylation, locking CAFs into a glycolytic state that persists after reoxygenation [22]. Whether analogous epigenetic commitment occurs in HNSCC stroma has not been tested, but the finding raises the possibility that once established, glyCAFs may remain stable even after the initiating signals are removed.

### Maintenance of metabolic coupling

Once established, the glyCAF phenotype is maintained through reciprocal signaling between CAFs and tumor cells rather than in isolation. Kumar et al. identified a bidirectional communication circuit that stabilizes metabolic coupling in HNSCC [14]. Using Seahorse extracellular flux analysis, they showed that CAF-secreted hepatocyte growth factor (HGF) increases glycolytic capacity and extracellular lactate production in HNSCC cells through activation of c-Met. In return, HNSCC cells secrete basic fibroblast growth factor (bFGF), which binds fibroblast growth factor receptor (FGFR) on CAFs and promotes further HGF secretion, creating a self-reinforcing metabolic circuit (Fig. 1B). Functional experiments also showed that CAFs efficiently utilized lactate as a carbon source, suggesting that CAFs can participate in metabolic exchange within the TME. Pharmacologic inhibition of c-Met and FGFR significantly reduced CAF-induced HNSCC tumor growth (*p* < 0.001), demonstrating that this signaling loop is therapeutically actionable. Together, the reciprocal HGF/c-Met and bFGF/FGFR axis provides a mechanism by which glyCAF-tumor metabolic coupling can be sustained over time and identifies a direct therapeutic bridge to clinical agents in development, including ficlatuzumab, an anti-HGF monoclonal antibody in Phase III evaluation for human papillomavirus (HPV)-negative recurrent or metastatic HNSCC [25].

## Functional consequences of GlyCAF metabolism

### Metabolic coupling and the lactate shuttle

The central functional consequence of the glyCAF phenotype is lactate export into the extracellular space, where it serves as a metabolic substrate for adjacent tumor cells (Fig. 1B). In HNSCC, reciprocal expression of MCT4 in stromal fibroblasts and MCT1 in proliferative carcinoma cells establishes a metabolic symbiosis, in which glyCAF-derived lactate is exported by fibroblasts and subsequently imported by tumor cells to fuel mitochondrial oxidative metabolism [13]. Imported lactate is converted back to pyruvate by lactate dehydrogenase B (LDHB) and enters the TCA cycle, supporting both oxidative phosphorylation and anabolic biosynthesis (Fig. 1B) [12]. By using lactate rather than glucose as a primary oxidative substrate, tumor cells can spare glucose for biosynthetic pathways and for glycolysis-dependent tumor cell subsets in hypoxic regions, enhancing overall bioenergetic resilience [12]. The prognostic relevance of this metabolic architecture has been confirmed in independent cohorts. Simões-Sousa et al. assessed MCT expression in oral cavity tumors and found that MCT4 expression in both tumor and stromal compartments was associated with aggressive disease features [26]. Leu et al. demonstrated that membranous MCT1 expression in HNSCC patients treated with radiochemotherapy was associated with clinical outcome, supporting the functional significance of lactate import capacity in this disease [27].

### Tumor progression phenotypes

Beyond metabolic fueling, glyCAF-derived lactate contributes to several tumor progression phenotypes. Lactate imported through MCT1 in endothelial cells activates reactive oxygen species production, which stimulates NF-κB signaling and induces vascular endothelial growth factor expression, promoting angiogenesis. Végran et al. demonstrated that inhibiting MCT1 in endothelial cells reduced both lactate-induced angiogenesis and tumor growth in vivo [28]. Elevated intratumoral lactate concentrations are also associated with metastatic potential. Brizel et al. measured lactate concentrations in HNSCC biopsies by bioluminescence imaging and found that high lactate levels predicted the development of metastatic disease and reduced overall survival [29]. Walenta et al. reported a similar correlation between lactate concentration and metastasis incidence in cervical cancers, suggesting that the prognostic significance of intratumoral lactate extends across solid tumor types [30]. Lactate utilization also appears linked to stem-like metabolic programs. Curry et al. demonstrated that in normal oral mucosa, MCT1 expression is highly selective for the basal epithelial stem cell layer, which is hyper-proliferative and enriched in mitochondrial markers [13]. In HNSCC tumors, a distinct carcinoma cell population exhibited the same metabolic phenotype (Ki-67^+^/TOMM20^+^/COX^+^/MCT1^+^), raising the possibility that lactate-fueled oxidative metabolism supports stem-like tumor cell populations associated with disease recurrence [13].

### Immune consequences

**Fig. 2:**
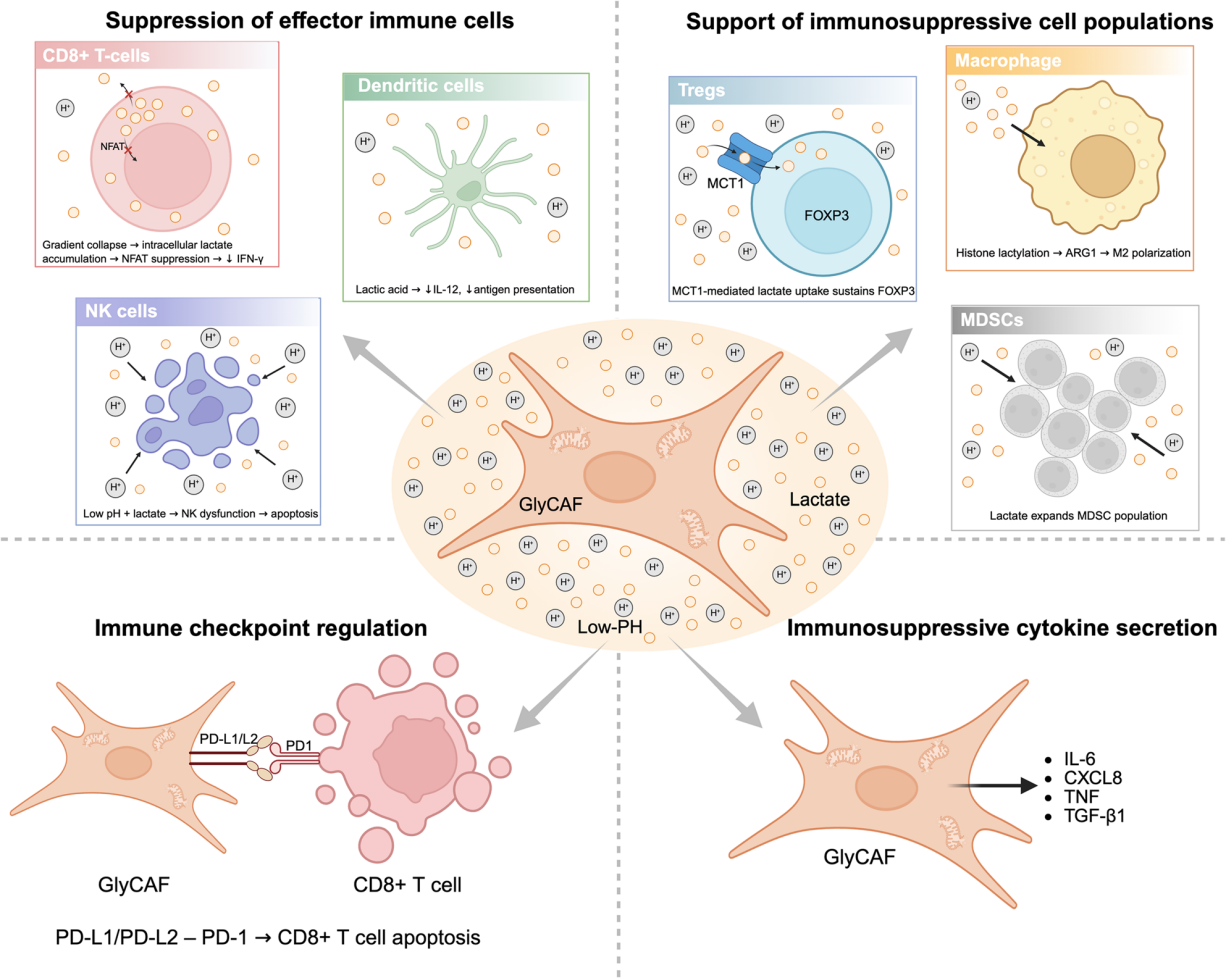
Immune-related functions of glyCAFs. GlyCAFs suppress effector immune cells, including CD8^+^ T cells, dendritic cells, and NK cells, through lactate accumulation and extracellular acidification. Concurrently, glyCAFs promote the expansion and activity of immunosuppressive populations such as regulatory T cells, tumor-associated macrophages, and myeloid-derived suppressor cells. These effects are further reinforced by glyCAF-associated enhancement of PD-L1/PD-L2-PD-1 signaling and the secretion of immunomodulatory cytokines, collectively fostering an immune-evasive TME

The accumulation of lactate in the TME and the associated extracellular acidification suppress the function of effector immune cells while simultaneously supporting immunosuppressive populations (Fig. 2). The effects on CD8 + cytotoxic T lymphocytes are among the best characterized. Fischer et al. demonstrated that lactic acid suppresses the proliferation and cytokine production of human cytotoxic T lymphocytes in a concentration-dependent manner, reducing cytotoxic activity by approximately 50% [31]. Mechanistically, high extracellular lactic acid concentrations impair lactate export from T cells by collapsing the transmembrane lactate gradient; this intracellular lactate accumulation disrupts T cell metabolism and function [31]. Brand et al. extended these findings in vivo, demonstrating that LDHA-derived lactic acid production in melanoma cells inhibits tumor immunosurveillance by T and natural killer (NK) cells [32]. In immunocompetent mice, tumors with reduced lactic acid output grew significantly slower and showed increased infiltration by IFN-γ-producing T and NK cells, while this growth difference was abolished in immunodeficient mice. Pathophysiological concentrations of lactic acid prevented upregulation of nuclear factor of activated T cells (NFAT) in both T and NK cells, providing a mechanistic basis for the suppression of IFN-γ production. Natural killer cells are further affected by lactate-mediated acidification. Harmon et al. showed that acidification induces apoptosis of liver-resident NK cells in a colorectal liver metastasis model, demonstrating that lactate can directly eliminate rather than merely suppress NK cell populations [33]. Husain et al. showed that tumor-derived lactate expands myeloid-derived suppressor cells while impairing NK cell function [34]. Dendritic cell function is also compromised: Gottfried et al. demonstrated that tumor-derived lactic acid alters dendritic cell antigen expression and reduces IL-12 secretion, indicating that the lactate-rich microenvironment impairs the ability of dendritic cells to prime effective anti-tumor T cell responses [35]. In addition to these well-characterized adaptive and innate immune populations, recent studies have identified group 2 innate lymphoid cells (ILC2s) as emerging anti-tumor immune regulators that are also suppressed within the lactate-rich TME [36]. Wagner et al. demonstrated that tumor-derived lactic acid inhibited proliferation and cytokine production and decreased the survival of ILC2s [37]. Melanomas with reduced LDHA expression exhibited delayed growth and increased ILC2 infiltration, and upon IL-33 stimulation, ILC2s accompanied by eosinophils more effectively restrained the growth of LDHA-low tumors than control melanomas. Database analysis further revealed a negative correlation between LDHA expression and markers associated with ILC2s in human cutaneous melanoma. These findings are consistent with the broader suppressive effects of lactic acid on innate lymphoid cell function, including inhibition of NFAT activity, which coordinates immune responses across multiple ILC subsets [38]. In pancreatic ductal adenocarcinoma, Moral et al. demonstrated that ILC2s activate tissue-specific anti-tumor immunity through the IL-33/ILC2 axis by recruiting dendritic cells and priming CD8^+^ T cells, and that PD-1 blockade relieves cell-intrinsic PD-1 inhibition to expand tumor ILC2s and enhance tumor control [39]. These studies demonstrate that lactate accumulation in the TME broadly compromises anti-tumor immunity across both adaptive and innate effector populations, reinforcing the immunosuppressive landscape shaped by glyCAFs.

In contrast to its suppressive effects on effector cells, lactate supports immunosuppressive populations. Watson et al. demonstrated that tumor-infiltrating regulatory T cells rely on MCT1-mediated lactate uptake and intracellular lactate metabolism to sustain FOXP3 expression and maintain their suppressive activity within the low-glucose, high-lactate TME [40]. Lactate also influences macrophage gene expression through histone lactylation, a novel epigenetic modification described by Zhang et al. in which lactate-derived lactyl groups are added to histone lysine residues, driving expression of homeostatic genes including ARG1 and promoting M2-like transcriptional programs [41]. Extending this epigenetic mechanism from macrophages to CAFs, Ye et al. demonstrated that glycolysis-derived lactate in CAFs induces H3K18 lactylation at the promoter regions of CXCL16 and its transcription factor FOXO3, upregulating CXCL16 expression and recruiting immunosuppressive TNFR2^+^ Treg cells via the CXCL16/CXCR6 axis [42]. Notably, glyCAF-mediated immune modulation also operates through glycolysis-dependent but not exclusively lactate-dependent mechanisms: Broz et al. showed that glyCAFs rely on GLUT1-dependent CXCL16 expression to exclude cytotoxic T cells from the tumor parenchyma in soft-tissue sarcomas, and that targeting glycolysis reduced glyCAF accumulation at the tumor margin and improved chemotherapy efficacy [43]. Beyond lactate and chemokine signaling, CAFs also supply glutamine to tumor cells through glutamine synthetase-mediated synthesis and autophagy-dependent release, establishing glutamine as an additional non-lactate metabolic currency in CAF-tumor coupling [44].

### HNSCC-specific CAF immune biology

The general principles of lactate-mediated immune suppression are further reinforced by CAF-intrinsic immunomodulatory programs in HNSCC. Takahashi et al. established matched CAF and normal fibroblast pairs from HNSCC patients and demonstrated that CAFs express elevated levels of the immune checkpoint ligands PD-L1 (B7-H1) and PD-L2 (B7-DC) compared to normal fibroblasts [45]. In co-culture assays, CAFs suppressed T cell proliferation, induced T cell apoptosis, and expanded regulatory T cell populations to a significantly greater degree than normal fibroblasts. The CAF secretome was enriched in immunosuppressive and proangiogenic factors including IL-6, CXCL8, TNF, TGF-β1, and VEGFA (Fig. 2). Li et al. added a spatial dimension to these findings through integrated single-cell and spatial transcriptomics on HNSCC specimens, identifying a CAF subset characterized by high expression of major histocompatibility complex class I (MHC-I) genes, galectin-9, and interferon-responsive signatures that forms organized stromal barriers physically restricting CD8^+^ T cell infiltration into tumor nests [46]. The abundance of this CAF subset inversely correlated with progenitor-like cytotoxic T cell populations, demonstrating that CAFs in HNSCC do not merely secrete immunosuppressive factors but actively organize the spatial architecture of the microenvironment in ways that exclude effector immune cells. Further evidence for the immune-modulatory significance of CAF heterogeneity comes from Obradovic et al., who demonstrated that specific CAF subtypes were immunostimulatory and predicted favorable response to anti-programmed cell death protein 1(PD-1) therapy, while other subtypes were immunosuppressive and associated with treatment resistance, suggesting that the composition of the CAF compartment, and not just its overall abundance, determines immunotherapy efficacy [7].

## Spatial organization of GlyCAF-tumor interactions

**Fig. 3:**
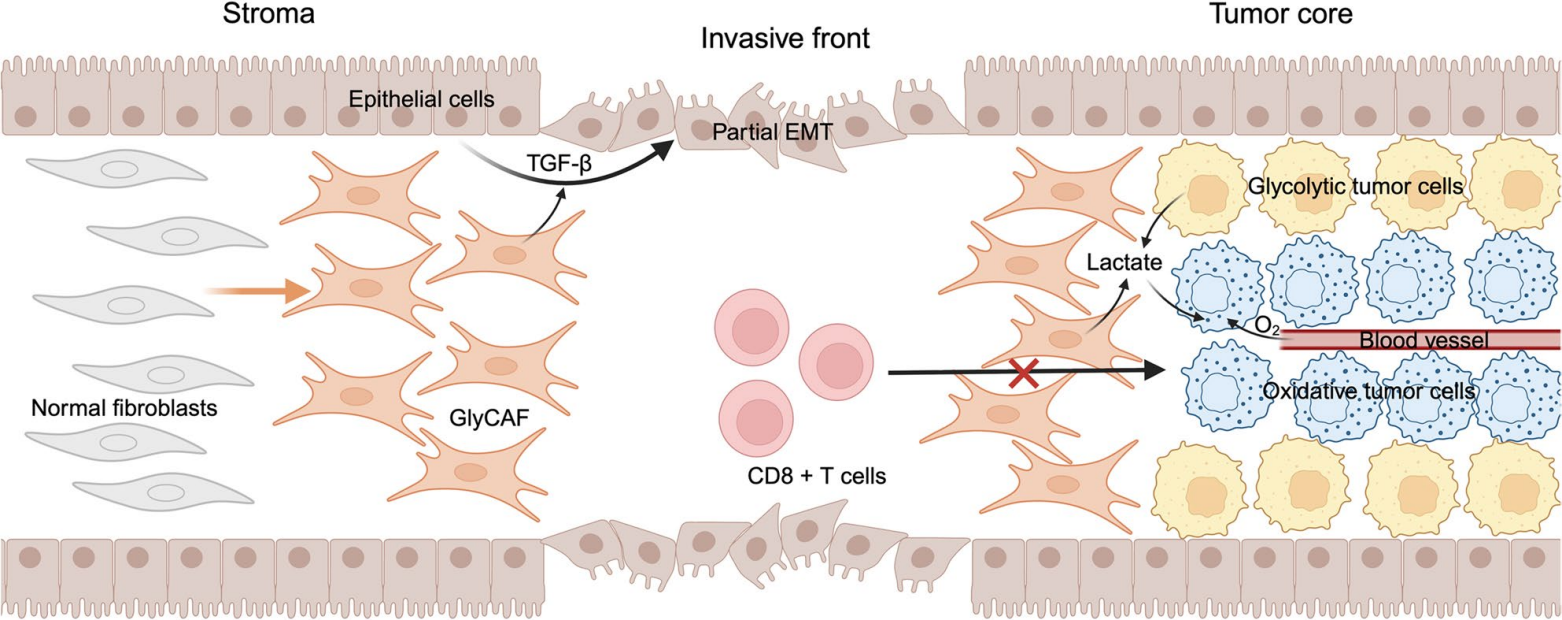
Spatial organization of glyCAFs in the TME. GlyCAFs display a spatially organized distribution within tumors, with progressive enrichment from the surrounding stroma toward the invasive front and tumor core. At the invasive front, glyCAFs are closely associated with TGF-β signaling and features of partial epithelial-mesenchymal transition, supporting local invasion and tissue remodeling. In this region, glyCAFs also contribute to the formation of a stromal barrier that limits CD8⁺ T cell infiltration into tumor nests. Within the tumor core, glyCAFs localize near cancer cells, where both glyCAFs and glycolytic tumor cells serve as major sources of lactate. This lactate is utilized by neighboring oxidative tumor cells, which are preferentially positioned near blood vessels and oxygen-rich regions, reinforcing metabolic compartmentalization within the TME

The spatial organization of the TME in HNSCC is not random but reflects structured interactions between glyCAFs and tumor cells, particularly at the invasive front where metabolic coupling, epithelial plasticity, and immune exclusion converge (Fig. 3). Understanding this architecture is essential because metabolic symbiosis depends on spatial proximity between lactate-producing stroma and lactate-consuming tumor cells, and the spatial distribution of glyCAFs relative to tumor epithelium is organized into functionally relevant metabolic zones.

Curry et al. documented that MCT4-positive stromal fibroblasts are concentrated in the tumor stroma adjacent to the invasive carcinoma front, while MCT1-positive, mitochondria-rich carcinoma cells localize to the proliferative compartments at the tumor leading edge [13]. This spatial arrangement suggests that metabolic symbiosis is most active at the invasive front, precisely where energy demand is highest and the tumor interfaces with surrounding normal tissue. Puram et al. provided complementary evidence from the transcriptomic perspective, demonstrating that malignant cells at the leading edge adopt a partial epithelial-to-mesenchymal transition (p-EMT) program that is spatially associated with the adjacent CAF population [5]. Ligand-receptor analysis suggested that CAF-secreted TGF-β and other signals induce this p-EMT program in nearby carcinoma cells (Fig. 3). The p-EMT signature was a strong predictor of nodal metastasis and adverse pathologic features, indicating that the CAF-tumor interface at the leading edge is a critical determinant of clinical outcome.

Arora et al. extended these observations using integrative single-cell and spatial transcriptomics applied to HPV-negative oral squamous cell carcinoma [47]. They demonstrated that the tumor core and leading edge are characterized by unique transcriptional profiles, neighboring cellular compositions, and ligand-receptor interaction networks. ECM-remodeling myofibroblastic CAFs (myoCAFs) exhibited substantially more interactions with cancer cells at the leading edge than cancer cell-cancer cell signaling in either compartment, highlighting a critical role for CAFs in modulating cancer cell behavior at the invasive front. The leading edge gene signature was associated with worse clinical outcomes across multiple cancer types, while the tumor core signature was associated with improved prognosis, reinforcing the idea that the invasive front represents a uniquely aggressive niche shaped by stromal contributions. As discussed above, the barrier-forming CAF subset identified by Li et al. inversely correlated with cytotoxic T cell infiltration [45], linking glyCAF spatial organization directly to immune exclusion at the invasive front.

While glyCAFs are concentrated at the invasive front, they coexist with other spatially distinct CAF subtypes. Biffi et al. demonstrated that tumor-secreted TGFβ and IL-1 establish spatially segregated CAF niches: TGFβ promotes myofibroblastic differentiation in tumor-adjacent fibroblasts, while IL-1 activates JAK/STAT signaling in distally located fibroblasts to generate inflammatory CAFs (iCAFs) [48]. These two states are interconvertible depending on spatial context. As iCAF-derived IL-6 activates STAT3 signaling, iCAFs may contribute to sustaining the glycolytic phenotype in neighboring fibroblasts. Elyada et al. identified antigen-presenting CAFs (apCAFs) expressing MHC class II molecules capable of activating CD4^+^ T cells but lacking costimulatory molecules [4]. Chen et al. further resolved two spatially distinct apCAF populations: mesothelial-like apCAFs near cancer cells and fibrocyte-like apCAFs associated with lymphocyte-enriched niches [49]. As apCAFs lack costimulatory molecules, they may promote regulatory T cell differentiation, potentially reinforcing the immunosuppressive microenvironment established by glyCAFs.

Whether glyCAFs are selectively enriched during disease progression remains an open question, though several lines of evidence suggest this is the case. Stromal MCT4 expression correlates with higher tumor stage, intratumoral lactate concentrations predict metastatic spread, and the lysyl oxidase (LOX)-positive inflammatory CAF subset identified by Liu et al., which overlaps with features attributed to glyCAFs, is predictive of poor prognosis and immunotherapy resistance [8]. However, these observations derive from independent datasets using different markers and analytic frameworks. No study has directly compared glyCAF abundance across matched premalignant lesions, early-stage primary tumors, advanced primary tumors, and lymph node metastases within the same cohort. Such a study, ideally employing multiplexed spatial profiling with metabolic markers on tissue microarrays spanning the disease continuum, would clarify whether glyCAF enrichment is an early event, a progression-associated phenomenon, or a prerequisite for metastatic dissemination.

## GlyCAFs as systems-level organizers of the TME

The preceding sections have examined the molecular identity of glyCAFs, the mechanisms governing their induction and maintenance, their functional consequences for tumor metabolism and immune regulation, and their spatial organization within the tumor architecture. Considered individually, each of these dimensions provides important insight into stromal biology. Considered together, they reveal a more fundamental role: glyCAFs operate as systems-level organizers of the HNSCC TME, coordinating tumor behavior across three coupled domains: metabolic fueling, immune suppression, and spatial structuring. These three domains are not independent. Lactate exported by glyCAFs through MCT4 simultaneously serves as an oxidative fuel for adjacent tumor cells, acidifies the extracellular space to suppress effector immune populations, and sustains immunosuppressive cells including regulatory T cells and M2-polarized macrophages. This metabolic output is spatially concentrated at the invasive front, where glyCAFs form organized stromal barriers that restrict CD8 ^+^ T cell access to tumor nests while maintaining proximity to MCT1-positive tumor cells exhibiting partial EMT. The result is a self-reinforcing niche in which metabolic symbiosis, immune evasion, and invasive behavior are not separate processes but coupled outputs of a single stromal population within a shared microenvironmental niche.

This integrated view positions glyCAFs not merely as metabolic fuel providers, as the original reverse Warburg model proposed [11], but as architects of the immunosuppressive and invasion-permissive microenvironment. This distinction matters because it shifts the therapeutic calculus. If glyCAFs only provided lactate, then blocking lactate transport might be sufficient. But if glyCAFs simultaneously organize immune exclusion and support epithelial plasticity at the invasive front, then effective intervention may require disrupting multiple glyCAF functions or targeting the signaling circuits that maintain glyCAF identity itself. This framework further generates a testable clinical prediction: tumors with high glyCAF density, identifiable through stromal MCT4 expression, are predicted to exhibit reduced responsiveness to immune checkpoint inhibitors compared to tumors with low glyCAF density. The rationale is that glyCAF-derived lactate impairs the metabolic fitness of T cells that encounter checkpoint signals, while the spatial exclusion of effector cells by organized CAF barriers limits the ability of checkpoint blockade to unleash anti-tumor immunity even when T cells are present within the broader tumor. Lactate-mediated support of regulatory T cells may further counteract the effects of checkpoint blockade by maintaining local immunosuppressive tone. While this prediction has not been tested in prospective clinical studies, it is consistent with the observation by Obradovic et al. that immunosuppressive CAF subtypes predict nivolumab resistance in HNSCC [7], and with the broader association between stromal MCT4 expression and advanced tumor stage reported by Curry et al. [13].

The systems-level model also reframes the relationship between glyCAFs and existing CAF classification schemes. Rather than proposing glyCAFs as an additional transcriptomic subtype alongside myofibroblastic, inflammatory, and antigen-presenting CAFs, we propose that glycolytic metabolism represents an orthogonal functional axis that intersects with and modulates these transcriptional identities. MyoCAFs that are also glycolytic may have fundamentally different effects on the microenvironment than one that is not. Incorporating metabolic state into CAF classification, whether through composite immunohistochemical panels, metabolic flux profiling, or integrated single-cell multi-omic approaches, would add a dimension that current transcription-based taxonomies lack and that may prove more predictive of clinical behavior than transcriptional identity alone.

Importantly, not all CAFs are tumor-promoting. Fibroblasts expressing FSP1 have been shown to protect the host from carcinogens through collagen production and encapsulation, and their elimination induced rapid tumor development [50]. Recent work has characterized two functionally distinct CAF populations: cancer-promoting CAFs (pCAFs) and cancer-restraining CAFs (rCAFs) [3]. Meflin-positive rCAFs in pancreatic ductal adenocarcinoma correlated with favorable patient outcomes, and their genetic ablation led to significant disease progression [51]. rCAFs have also been identified in oral carcinoma, where a CAF subtype with lower α-smooth muscle actin expression suppresses stemness through BMP4 [52], and in intestinal tumors, where IKKβ acts as a tumor suppressor in CAFs [53]. Notably, rCAFs initially protect normal tissues from tumor cell invasiveness, but as disease progresses, tumor cells may reprogramme them to establish a stromal environment that nurtures tumor development [3, 54]. This functional duality reinforces the rationale for metabolic reprogramming of glyCAFs rather than indiscriminate fibroblast ablation, as therapeutic strategies must preserve the tumor-restraining capacity of specific CAF populations.

## Pan-cancer perspective

Although glyCAF research in HNSCC is still emerging, glycolytic CAFs have been characterized across multiple solid tumor types. Reviewing this literature identifies conserved features that strengthen the generality of the glyCAF framework and highlights cancer-type-specific variations that may inform context-dependent therapeutic strategies (Table 2). While lactate is the dominant metabolite in the glyCAF-tumor axis in HNSCC, other tumor types utilize alternative metabolic currencies, indicating that glyCAF-like metabolic support is conserved in principle but adapted in execution.

**Table 2:** Pan-cancer evidence for glyCAFs

Cancer type	Reprogramming signal(s)	Key glyCAF markers	Functional consequences	Prognostic significance
HNSCC	ROS/H₂O₂, ISG15/CD11a, HGF/bFGF loop, cigarette smoke, TGF-β	MCT4^+^, MCT1^−^, TOMM20-low, COX-low, IDH3α-low	Lactate shuttle to oxidative tumor cells; immune suppression; p-EMT induction at invasive front	Stromal MCT4 correlates with tumor stage; epithelial MCT4 predicts recurrence
Breast	Cav-1 loss, ROS, TGF-β, oxidized ATM, HIF1A hypomethylation	Stromal MCT4^+^, Cav-1 loss, GLUT1^+^	4-fold tumor mass increase with glycolytic fibroblasts; 10-fold lung metastases with lactate	Stromal MCT4: <18% vs. ~ 97% 10-yr survival in TNBC; Cav-1 loss: 3.6-fold reduction in PFS
Prostate	Direct contact, SIRT3/HIF-1α normoxic stabilization	MCT4^+^, GLUT1^+^; tumor cells MCT1^+^	Mitochondrial transfer via nanotubes; SIRT1/PGC-1α axis; PKM2 nuclear translocation and EMT; CD4^ +^ T cell polarization to Tregs	High stromal MCT4 and epithelial MCT1 predicts poor outcome (*n* = 480)
Pancreatic	Autophagy-dependent reprogramming; co-culture-induced glycolysis	MCT4^+^; alanine secretion; meCAFs with active glycolysis	Alanine shuttle via SLC38A2; shikonin overcomes gemcitabine resistance	meCAF-rich tumors: 64.7% ORR to anti-PD-1 vs. poor response in myCAF-dominant
Gastric	Cancer cell metastatic potential determines fibroblast reprogramming degree	Stromal MCT4^+^, epithelial TOMM20^+^, Cav-1 loss	Highly metastatic cells strongly induce glycolysis genes in fibroblasts; low-metastatic cells do not	Stromal MCT4: independent predictor of OS (HR = 1.96) and DFS (HR = 2.08); Cav-1 loss predicts poor DFS and OS
Ovarian	LPA/LPAR/HIF-1α; CXCL14/LINC00092/PFKFB2; TGF-β1/p38α/CCL5	Glycolytic shift precedes α-SMA; PFKFB2^+^	LPA as earliest CAF priming event; lncRNA-driven metabolic feedback loop; glycogenolysis mobilization	LINC00092/PFKFB2 silencing inhibits metastasis in vivo
Colorectal	Nrf2-regulated reciprocal MCT expression	Stromal MCT4^+^, epithelial MCT1^+^, Cav-1 loss	Reverse Warburg metabolic phenotype with reciprocal transporter expression	Stromal Cav-1 loss independently predicts poor DFS and OS (*n* = 178)
Lung adenocarcinoma	TGF-β-driven GFPT2 upregulation; ROS-dependent bidirectional reprogramming	GFPT2/GFAT2^+^ (hexosamine pathway); CAF-expressed FDG-PET genes	FDG-PET glucose uptake genes expressed by CAFs, not malignant cells (adenocarcinoma-specific; reversed in SCC)	GFAT2 in fibroblasts predicts worse 5-yr survival (HR = 2.05)

### Conserved features

Across different types of solid tumors, tumor cells reprogram adjacent fibroblasts into a glycolytic state that supports metabolic symbiosis with oxidative tumor cells [11, 55–58]. Tumor-derived signals including reactive oxygen species, TGF-β, or direct cell contact drive HIF-1α-dependent metabolic reprogramming in stromal fibroblasts, enabling MCT4-mediated metabolite export and uptake by tumor cells through MCT1 [11]. Stromal MCT4 expression and loss of stromal Caveolin-1 serve as prognostic biomarkers across multiple tumor types, with clinical outcome data consistently associating these markers with aggressive disease features and reduced survival [55, 56, 59–61].

### Cancer-type-specific variations

The glyCAF concept originated in breast cancer, where Pavlides et al. first described the reverse Warburg effect and where clinical validation is strongest [11]. Witkiewicz et al. demonstrated that in triple-negative breast cancer, high stromal MCT4 correlated with less than 18% survival at 10 years, while absent stromal MCT4 predicted approximately 97% survival; critically, epithelial MCT4 had no prognostic value, and only the stromal compartment mattered [55]. Bonuccelli et al. showed that glycolytic fibroblasts co-injected with breast cancer cells produced a 4-fold increase in tumor mass and that lactate injections produced an approximately 10-fold increase in lung metastases [62]. Sun et al. identified that hypoxia activates oxidized ATM in breast CAFs to enhance glycolysis and tumor invasion [63], and Becker et al. further showed epigenetic stabilization of the glycolytic state through HIF1A promoter hypomethylation [22].

In prostate cancer, the Chiarugi group systematically characterized metabolic symbiosis between CAFs and tumor cells. Fiaschi et al. demonstrated that intercellular contact triggers a glycolytic switch in fibroblasts via SIRT3-dependent HIF-1α stabilization under normoxic conditions, and that MCT1 inhibition dramatically impaired prostate cancer cell survival and tumor outgrowth in vivo [19]. Subsequent studies revealed additional downstream consequences: CAF-derived lactate alters the NAD^+^/NADH ratio in cancer cells, activating a SIRT1/PGC-1α axis that enhances mitochondrial mass, and cancer cells hijack functional mitochondria from CAFs through tunneling nanotubes [64]. Giannoni et al. demonstrated that CAFs promote nuclear PKM2 translocation in cancer cells, forming a complex with HIF-1α that fosters EMT, and that the PKM2 activator DASA-58 reduced lung metastases approximately 6-fold [65]. Comito et al. showed that CAF-derived lactate polarizes CD4^+^ T cells toward an immunosuppressive phenotype through SIRT1-mediated T-bet degradation while expanding Tregs [66]. Clinical validation from Pértega-Gomes et al. in 480 prostate cancer tissues confirmed that high stromal MCT4 combined with strong epithelial MCT1 predicted poor outcome [59].

Pancreatic ductal adenocarcinoma presents distinctive glyCAF biology shaped by its uniquely hypovascular, nutrient-poor microenvironment. Sousa et al. demonstrated that pancreatic stellate cells undergo autophagy-dependent metabolic reprogramming, secreting alanine rather than lactate as a primary fuel for tumor cells [67]. Using isotope tracing, they showed that alanine outcompeted glucose and glutamine-derived carbon in fueling the TCA cycle, shifting the metabolite currency of stromal-epithelial coupling. Parker et al. subsequently identified SLC38A2 as the specific alanine transporter, and its loss produced significant tumor regression in xenograft models [68]. The classical lactate-based paradigm also operates in pancreatic cancer: shikonin suppressed MCT4 expression and overcame CAF-induced gemcitabine resistance in vivo [69]. Single-cell RNA sequencing has revealed metabolic CAFs (meCAFs) displaying highly active glycolysis, and patients with meCAF-rich tumors showed a 64.7% objective response rate to immunotherapy compared to poor responses in myoCAF-dominant tumors, providing direct evidence that glycolytic CAF subtypes can predict immunotherapy efficacy [70]. This observation offers the closest existing clinical support for the prediction outlined in Sect. 6 stating that glyCAF density may predict response to immune checkpoint blockade and suggests that similar biomarker-guided stratification could be applied in HNSCC.

In gastric cancer, clinical evidence for the reverse Warburg effect is strong. Stromal MCT4 and epithelial TOMM20 were positively correlated and both served as independent prognostic factors for reduced overall and disease-free survival across multiple cohorts [56], and low Cav-1 in CAFs independently predicted poor outcomes [60]. An important mechanistic insight came from Kogure et al., who demonstrated that cancer cell metastatic potential determines the degree of fibroblast glycolytic reprogramming: highly metastatic gastric cancer cells strongly induced glycolysis-related gene upregulation in co-cultured fibroblasts, while low-metastatic cells did not, suggesting that the aggressiveness of the tumor dictates the metabolic phenotype of its stroma [71].

Ovarian cancer reveals unique mechanisms centered on lipid signaling. Radhakrishnan et al. demonstrated that lysophosphatidic acid, abundant in ovarian cancer ascites, induces a glycolytic shift in normal fibroblasts as the earliest priming event in the fibroblast-to-CAF transition, preceding α-SMA expression by days [72, 73]. Zhao et al. identified a long non-coding RNA (lncRNA)-driven metabolic feedback loop in which CAF-derived CXCL14 upregulates LINC00092 in ovarian cancer cells, which directly binds the glycolytic enzyme PFKFB2, increasing glycolysis; the glycolytic cancer cell phenotype reciprocally sustains CAF-like features in fibroblasts [73]. Curtis et al. described a distinct mechanism in which CAFs mobilize tumor cell glycogen stores through TGF-β1-activated p38α MAPK signaling, with glycogen phosphorylase inhibition reducing metastasis in vivo [74].

In colorectal cancer, Diehl et al. demonstrated reciprocal MCT1 (epithelial) and MCT4 (stromal) expression regulated by Nrf2 [57], and Zhao et al. showed that loss of stromal Cav-1 independently predicted poor survival while tumor cell Cav-1 showed no clinical correlation [61]. Lung cancer presents a striking histology-dependent pattern: Zhang et al. discovered that in lung adenocarcinoma, genes correlating with FDG-PET glucose uptake were predominantly expressed by CAFs rather than malignant cells, and identified GFPT2 as a key CAF-expressed gene driving the hexosamine biosynthesis pathway, with GFAT2 protein in fibroblasts predicting worse 5-year survival [58]. This pattern was specific to adenocarcinoma; in squamous cell carcinoma, glucose uptake was driven by malignant cells themselves, demonstrating that the relative metabolic contribution of stroma versus tumor is histology dependent.

### Cross-cancer synthesis

Across these cancer types, the core architecture of glyCAF-tumor metabolic coupling is conserved, with stromal metabolic reprogramming recurrently supporting oxidative tumor cell metabolism through compartmentalized metabolite exchange. Stromal MCT4 and Cav-1 loss serve as prognostic biomarkers across multiple malignancies. However, each cancer type contributes unique biology (Table 2): breast cancer established the Cav-1 loss paradigm with the strongest clinical validation; prostate cancer revealed SIRT3-mediated reprogramming and mitochondrial transfer through nanotubes; pancreatic cancer shifted the metabolite focus from lactate to alanine and introduced glycolytic CAF subtypes as immunotherapy biomarkers; and lung adenocarcinoma highlighted the hexosamine biosynthesis pathway as a CAF-specific metabolic driver. These parallels validate the generality of the glyCAF concept while the variations indicate that therapeutic strategies targeting glyCAF-tumor coupling will likely need to be adapted to the specific metabolic currency and induction mechanisms operating in each tumor type. These pan-cancer parallels provide a rationale for therapeutic intervention: if glyCAF-tumor metabolic coupling is conserved and clinically consequential across tumor types, then disrupting it represents a broadly applicable strategy.

## Therapeutic targeting of GlyCAF-tumor coupling

**Fig. 4:**
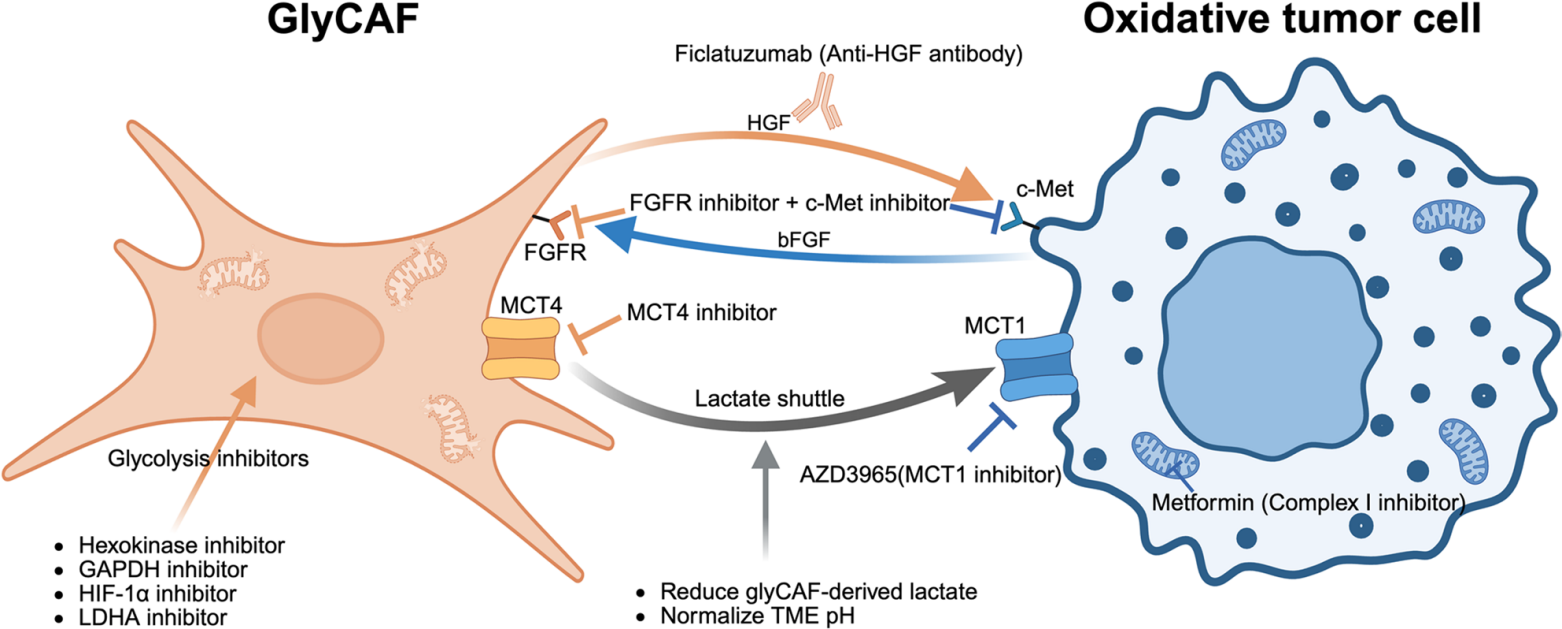
Therapeutic targeting of glyCAF-tumor metabolic coupling. Targeted therapeutic strategies can be deployed at multiple nodes along the glyCAF-tumor metabolic axis. These include inhibition of glycolysis within glyCAFs to limit lactate production, blockade of MCT4-dependent lactate export from glyCAFs, and disruption of MCT1-mediated lactate uptake by tumor cells. Targeting tumor oxidative metabolism, for example with metformin, further interferes with this metabolic coupling. In parallel, combination approaches aimed at interrupting reciprocal paracrine signaling, such as the HGF-c-MET and bFGF-FGFR axes, can disrupt bidirectional communication between glyCAFs and tumor cells. Additional lactate-directed strategies seek to reduce glyCAF-derived lactate accumulation and restore physiological pH within the TME, eventually alleviating metabolic and immune suppression

Therapeutic strategies targeting glyCAF-tumor metabolic coupling can be conceptualized as interventions at multiple levels: metabolite transport, stromal-tumor signaling, metabolic pathway dependency, and disruption of the physical stromal barrier (Fig. 4). Several of these strategies have entered clinical development, while others remain at the preclinical stage.

### Targeting lactate transport

The dependence of metabolic symbiosis on monocarboxylate transporter-mediated lactate flux makes MCTs attractive therapeutic targets. AZD3965 is a selective, orally bioavailable MCT1 inhibitor that has completed Phase I evaluation in patients with advanced solid tumors, establishing a recommended Phase 2 dose with primarily Grade 1–2 adverse events [75]. Preclinical studies have demonstrated that AZD3965 enhances the efficacy of fractionated radiotherapy in small cell lung cancer xenografts [76]. However, sensitivity to AZD3965 is inversely associated with MCT4 expression, which confers resistance by providing an alternative lactate efflux pathway [77, 78]. This resistance mechanism is particularly relevant in HNSCC, where glyCAFs predominantly express MCT4 rather than MCT1. As a result, AZD3965 primarily targets lactate uptake in tumor cells and disrupts the tumor cell component of the metabolic symbiosis, but does not directly inhibit lactate production and export by glyCAFs. While no selective MCT4 inhibitors have entered clinical trials, the genetic evidence of attenuated oral carcinogenesis in MCT4-knockout mice supports the therapeutic rationale [21]. Dual MCT1/MCT4 targeting strategies or selective MCT4 inhibitors represent a key unmet need in this field.

### Targeting stromal-tumor signaling

An alternative approach is to disrupt the upstream signaling pathways that maintain glyCAF-tumor metabolic coupling. The HGF/c-Met axis on tumor cells and the bFGF/FGFR axis on CAFs constitute the bidirectional signaling circuit identified by Kumar et al. that sustains the glyCAF phenotype [14]. Ficlatuzumab, an anti-HGF monoclonal antibody, has advanced furthest in HNSCC [79]. A Phase I trial of ficlatuzumab combined with cetuximab in cetuximab-resistant recurrent/metastatic HNSCC established a recommended Phase II dose with a clinical benefit rate of 67% [79]. A subsequent randomized Phase II trial demonstrated a progression-free survival benefit for the combination in HPV-negative recurrent/metastatic HNSCC [25]. Based on these results, the FIERCE-HN trial, a multicenter, randomized, double-blind, placebo-controlled Phase III study, is currently evaluating ficlatuzumab plus cetuximab versus placebo plus cetuximab in patients with recurrent/metastatic HPV-negative HNSCC, with overall survival as the primary endpoint [80]. While these trials were designed to overcome cetuximab resistance through dual epidermal growth factor receptor (EGFR)/HGF pathway inhibition rather than to specifically disrupt glyCAF-tumor metabolic coupling, the mechanistic overlap is significant: CAF-secreted HGF is the ligand that ficlatuzumab sequesters, and disruption of this axis would be expected to attenuate the signaling loop that maintains glyCAF identity. Combined c-Met and FGFR inhibition reduced CAF-induced tumor growth in preclinical models [14], suggesting that targeting both arms of the reciprocal loop may be more effective than targeting either alone.

### Targeting metabolic pathways and combination strategies

Direct inhibition of glycolytic metabolism within glyCAFs represents a third strategic approach. The glycolytic inhibitor 2-deoxyglucose competitively inhibits hexokinase, while 3-bromopyruvate primarily targets glyceraldehyde-3-phosphate dehydrogenase (GAPDH); both reduce glycolytic flux and lactate production [81]. While these agents lack cellular specificity, their combination with agents that selectively target oxidative metabolism could exploit the distinct metabolic dependencies of glyCAFs versus tumor cells. HIF-1α inhibitors could reduce glycolytic gene expression in CAFs, since HIF-1α is the master transcriptional regulator of glycolytic enzymes and MCT4 [18]. LDHA inhibitors, which would reduce lactate production at the terminal step of glycolysis, represent another approach, though clinically viable agents remain in early development.

Metabolic combination strategies may offer the most promising path forward. Beloueche-Babari et al. demonstrated that AZD3965 treatment increases mitochondrial metabolism in tumors as cells compensate for blocked lactate transport by shifting to oxidative pyruvate metabolism [82]. This adaptive response creates a vulnerability: combination with mitochondrial complex I inhibitors, including metformin, counteracted this compensatory shift and enhanced anti-tumor effects. The rationale is a metabolic double hit in which lactate transport is inhibited while the compensatory increase in oxidative phosphorylation is simultaneously constrained. Noble et al. confirmed that combination of AZD3965 with the mitochondrial complex I inhibitor BAY 87-2243 induced profound cell death in lymphoma models [78]. Reducing glyCAF-derived lactate before or concurrently with immune checkpoint blockade could reverse lactate-mediated immunosuppression and convert resistant tumors into responsive ones, directly addressing the clinical prediction outlined in Section "GlyCAFs as Systems-Level Organizers of the TME". However, normalizing the metabolic environment alone may be insufficient if organized CAF barriers continue to physically exclude effector T cells from tumor nests. Disrupting the stromal barrier may require complementary approaches such as TGF-β pathway inhibition, which maintains both the CAF barrier phenotype and p-EMT at the invasive front, or targeting ECM-remodeling programs including LOX activity in inflammatory CAFs. TGF-β pathway inhibition represents a complementary strategy, as TGF-β signaling sustains both the CAF barrier phenotype and p-EMT at the invasive front. Galunisertib, a small-molecule TGF-β receptor I kinase inhibitor, has been evaluated in Phase II trials in pancreatic and hepatocellular carcinoma [82], though not yet in HNSCC in combination with metabolic targeting agents. Whether metabolic reprogramming of glyCAFs itself weakens barrier integrity remains untested but represents an important question, since the metabolic and structural functions of these fibroblasts may be coupled rather than independent.

### Challenges and limitations

Several challenges must be addressed before metabolic targeting of glyCAFs can be translated to clinical practice. MCT1 is expressed in normal tissues including the retina and heart, as evidenced by the ocular and cardiac toxicities observed in the Phase I AZD3965 trial [75], and therapeutic windows may be narrow. Biomarker-guided patient selection, particularly identifying tumors with high MCT1 and low MCT4 expression, will likely be essential for MCT1-directed strategies [77]. MCT4-mediated resistance limits the efficacy of MCT1-selective inhibitors [78], and because glyCAFs express MCT4, MCT1 inhibitors target only the tumor cell side of the metabolic symbiosis; the development of selective MCT4 inhibitors or dual MCT1/MCT4 strategies will be necessary to address this limitation. Metabolic plasticity is a further concern: CAFs, like tumor cells, may adapt to glycolysis inhibition by activating alternative metabolic pathways including glutaminolysis, fatty acid oxidation, or enhanced mitochondrial function, and sustained metabolic targeting may require combination approaches that anticipate and block these adaptive responses. Finally, HNSCC-specific clinical data for metabolic targeting strategies remain limited. While ficlatuzumab trials are ongoing in HNSCC [80], trials directly testing MCT inhibition or biomarker-guided metabolic strategies in this disease have not been initiated, representing a critical gap that must be addressed to establish the translational utility of the glyCAF concept.

## Future directions

Current CAF classification systems do not account for metabolic state as a defining variable. It remains unresolved whether glyCAFs correspond to a discrete fibroblast population or instead represent a metabolic program that can emerge across multiple CAF subtypes. Resolving this question will require integrated analyses that combine single-cell transcriptomics with direct measurements of metabolic activity, including flux profiling, Seahorse assays, or emerging single-cell metabolomic platforms applied to matched specimens. Complementary spatial approaches, including multiplexed error-robust fluorescence in situ hybridization (MERFISH), CO-Detection by indEXing (CODEX), and multiplexed ion beam imaging with metabolic markers, could further define how glyCAFs are positioned relative to immune niches and proliferative tumor compartments.

The relationship between glyCAF abundance and disease progression remains unclear. Several independent lines of evidence suggest that glyCAFs are enriched in more advanced disease [8, 13, 29], but no study has directly compared glyCAF abundance across matched premalignant lesions, early-stage primary tumors, advanced primary tumors, and lymph node metastases within the same cohort. Such a study, employing multiplexed spatial profiling with metabolic markers on tissue microarrays spanning the disease continuum, would clarify whether glyCAF enrichment is an early event, a progression-associated phenomenon, or a prerequisite for metastatic dissemination. In addition, the impact of viral status on glyCAF biology remains largely unexplored. For example, HNSCC driven by HPV differ fundamentally from HPV-negative tumors in terms of TME composition, mutational landscape, immune contexture, and clinical behavior; however, how stromal metabolic organization varies between these subtypes, and specifically whether glyCAFs differ in abundance, molecular identity, signaling interactions, or functional roles, represents an important gap in knowledge. Notably, the major spatial and single-cell datasets generated (Puram et al. [5], Arora et al. [46], Li et al. [45]) focus primarily or exclusively on HPV-negative specimens, leaving glyCAF biology in HPV-positive HNSCC essentially uncharacterized.

Whether glyCAFs represent a stable fibroblast subtype or a reversible metabolic state remains an open question with important biological and therapeutic implications. Several lines of evidence support the view that the glycolytic phenotype is initially reversible. Cigarette smoke extract induces glycolytic reprogramming in previously quiescent fibroblasts, demonstrating that the transition is inducible and not a fixed lineage property [16]. In pancreatic cancer, all-trans retinoic acid (ATRA) restores quiescence in activated pancreatic stellate cells, reducing cancer cell proliferation and invasion [83], through RAR-β-dependent downregulation of actomyosin contractility [84]. These findings establish that activated, pro-tumorigenic fibroblast states can be pharmacologically reversed. However, evidence also supports progressive stabilization of the glycolytic phenotype. Becker et al. demonstrated that chronic hypoxia induces HIF1A promoter hypomethylation in breast cancer CAFs, locking in the glycolytic program even after reoxygenation [22]. This implicates that glyCAFs may exist along a continuum, from an initially reversible metabolic state to an epigenetically committed phenotype, with the degree of commitment depending on the duration and intensity of inducing signals. Resolving where HNSCC glyCAFs fall along this continuum will require longitudinal studies comparing early-stage and advanced tumors and will determine whether therapeutic strategies should aim to reprogram glyCAFs to a quiescent state or instead target their function and survival.

## Conclusions

The evidence reviewed here supports a model in which glyCAFs act as systems-level organizers of the TME, integrating metabolic support, immune modulation, and spatial structuring at the invasive front. This framework is supported by analyses of patient tissues, validated in genetic models such as MCT4 knockouts, and reinforced by a targetable reciprocal signaling network between glyCAFs and tumor cells. Future studies are needed to define how glyCAF abundance, molecular identity, and functional roles evolve with disease stage, viral status, and treatment, and to determine whether their glycolytic program can be therapeutically reprogrammed to a quiescent state rather than requiring complete ablation.

## Data Availability

No datasets were generated or analysed during the current study.

## Abbreviations

α-SMA: Alpha-smooth muscle actin
ATRA: All-trans retinoic acid
CAF: Cancer-associated fibroblast
CODEX: Co-detection by indexing
ECAR: Extracellular acidification rate
ECM: Extracellular matrix
EGFR: Epidermal growth factor receptor
EMT: Epithelial-mesenchymal transition
FDG-PET: Fluorodeoxyglucose positron emission tomography
FGF: Fibroblast growth factor
FGFR: Fibroblast growth factor receptor
glyCAF: Glycolytic cancer-associated fibroblast
HGF: Hepatocyte growth factor
HIF-1α: Hypoxia-inducible factor 1-alpha
HNSCC: Head and neck squamous cell carcinoma
HPV: Human papillomavirus
IC50: Half-maximal inhibitory concentration
ILC2s: Group 2 innate lymphoid cells
LDHA: Lactate dehydrogenase A
lncRNA: Long non-coding RNA
MCT1: Monocarboxylate transporter 1
MCT4: Monocarboxylate transporter 4
MERFISH: Multiplexed error-robust fluorescence in situ hybridization
MHC-I: Major histocompatibility complex class I
myoCAF: Myofibroblastic CAF
NF-κB: Nuclear factor kappa-light-chain-enhancer of activated B cells
NK: Natural killer
OCR: Oxygen consumption rate
OSCC: Oral squamous cell carcinoma
pCAFs: Cancer-promoting CAFs
PD-1: Programmed cell death protein 1
PD-L1: Programmed death-ligand 1
p-EMT: Partial epithelial-mesenchymal transition
rCAFs: Cancer-restraining CAFs
ROS: Reactive oxygen species
TGF-β: Transforming growth factor beta
TME: Tumor microenvironment
